# Evaluation of serum MIP-1β and MCP-2 levels in major depressive disorder: A case-control study

**DOI:** 10.1371/journal.pone.0305734

**Published:** 2024-06-18

**Authors:** Mariya Akter, A. S. M. Roknuzzaman, Mohammad Shahriar, Sardar Mohammad Ashraful Islam, Mohiuddin Ahmed Bhuiyan, M. M. A. Shalahuddin Qusar, Eva Rahman Kabir, Rabiul Islam

**Affiliations:** 1 Department of Pharmacy, University of Asia Pacific, Farmgate, Dhaka, Bangladesh; 2 Department of Psychiatry, Bangabandhu Sheikh Mujib Medical University, Shahabagh, Dhaka, Bangladesh; 3 School of Pharmacy, BRAC University, Merul Badda, Dhaka, Bangladesh; Post Graduate Institute of Medical Education and Research, INDIA

## Abstract

**Background:**

Major depressive disorder (MDD) is a common and debilitating mental illness characterized by persistent feelings of sadness, hopelessness, and a lack of interest in daily activities. The objective of this study was to investigate whether levels of macrophage inflammatory protein-1β (MIP-1β) and macrophage chemoattractant protein-2 (MCP-2) in the blood were associated with the pathophysiology and development of MDD compared to healthy controls (HCs).

**Methods:**

This case-control study was conducted involving 50 MDD patients and 38 HCs. We performed a comprehensive assessment to match age, sex, BMI, and socio-demographic profile between the groups. The study excluded participants with chronic infection, inflammatory diseases, coexisting psychiatric disorder, history of liver and kidney diseases, and individuals who are under antipsychotic medications. A professional psychiatrist diagnosed MDD patients and evaluated HCs based on the Diagnostic and Statistical Manual of Mental Disorders-5 (DSM-5) criteria. The severity of depression was assessed using the Hamilton Depression (Ham-D) rating scale. Commercially available enzyme-linked immunosorbent assay (ELISA) kits were used to quantify the serum MIP-1β and MCP-2 levels.

**Results:**

The results indicated elevated serum MIP-1β levels (207.73±24.24 pg/ml) in MDD patients compared to HCs (58.77±9.14 pg/ml). This difference in concentration is positively correlated with severity of disease symptoms (r = 0.451; p<0.001). Similarly, the levels of MCP-2 were found to be elevated in patients compared to controls (143.61±19.92 vs. 56.84±4.02 pg/ml; p = 0.003), with a positive correlation with the Ham-D scores (r = 0.373; p = 0.004).

**Conclusion:**

According to this study, elevated levels of MIP-1β and MCP-2 may be associated with the pathophysiology and development of MDD. These increased serum MIP-1β and MCP-2 levels could be used as risk assessment tools for MDD. The present findings urge further research and the development of therapeutic and diagnostic approaches for depression.

## 1. Introduction

Major depressive disorder (MDD), is a pervasive and debilitating mental health condition characterized by persistent feelings of sadness, despair, and a loss of interest or pleasure in daily activities [1–5]. MDD is a highly prevalent mental health disorder that affects people across all age groups, sexes, and socioeconomic backgrounds [5–7]. This poses a substantial public health challenge with wide-ranging consequences [6–8]. MDD imposes a significant burden, impacting the affected individuals and their communities, families, and healthcare systems [9–12]. A complex interplay of biological, neuroendocrine, neuroinflammatory, and structural alterations within the brain comprise the pathophysiology of MDD [13–15]. At the core of the neurobiological comprehension, MDD lies the dysregulation of neurotransmitter systems such as dopamine, serotonin, and norepinephrine-which are indispensable for the regulation of motivation, reward processing, and mood. Decreased serotonin levels and abnormalities in receptor sensitivity are frequent manifestations of serotonin system dysregulation in patients with MDD, which contribute to the development of depressive symptoms [16]. In addition, MDD is characterized by dysregulation of the hypothalamic-pituitary-adrenal (HPA) axis, which controls the body’s stress response [17]. Prolonged stress has the potential to induce hyperactivity of the HPA axis, leading to increased cortisol secretion [18]. This, in turn, could potentially exacerbate hippocampal atrophy, hinder neurogenesis, and cause disturbances in neurotransmitter functionality. In addition, the pathophysiology of MDD has been linked to neuroinflammatory processes [14], which are distinguished by increased concentrations of pro-inflammatory cytokines and activation of microglia. These processes disrupt neurotransmitter signaling, neuroplasticity, and the regulation of mood. Reductions in hippocampal volume and dysfunction of the prefrontal cortex have been identified in individuals with MDD via structural imaging studies, highlighting the role of structural changes in the manifestation of depressive symptoms. The development and progression of MDD are additionally influenced by environmental stressors, genetic predispositions, and genetic stressors [19, 20]. A comprehensive comprehension of the intricate pathogenesis of MDD is vital to develop diagnostic strategies and targeted interventions.

Traditionally, immune function, chemokines are becoming more and more associated in the pathophysiology of MDD. Research indicates that they influence neuroplasticity, contribute to neuroinflammation, and impact neurotransmitter systems associated with depression, although their precise role is still unknown [21–24]. In the serum and cerebrospinal fluid of MDD patients, elevated concentrations of chemokines such as interleukin-8 (IL-8/CXCL8), monocyte chemoattractant protein-4 (MCP-4), and normal T cells expressed and secreted (RANTES/CCL5) are detected [6, 25, 26]. Chemokines that stimulate microglia result in the secretion of pro-inflammatory cytokines, which sustain neuroinflammation that is associated with MDD. Furthermore, chemokines affect neuroplasticity by modifying neural circuits dedicated to mood regulation and synaptic plasticity [27]. Chemokines also interact with neurotransmitter systems such as serotonin, dopamine, and glutamate, which further alters the pathogenesis of MDD. Gaining insight into the function of chemokines presents encouraging prospects for therapeutic interventions in MDD.

Macrophage inflammatory protein-1β (MIP-1β), also known as CCL4, is a chemokine involved in immune regulation and inflammatory responses [28]. Recent studies have indicated that MIP-1β might be involved in the pathogenesis of MDD, specifically concerning mood dysregulation and neuroinflammation. Alterations in immune function and neuroinflammation have gained increasing interest as contributing factors to the development and progression of depressive symptoms [29–31]. It has been observed that individuals with MDD have elevated levels of the pro-inflammatory chemokine MIP-1β in both peripheral blood and central nervous system (CNS) tissues in comparison to healthy controls (HCs) [29, 30]. This implies that MIP-1β might play a role in mediating inflammatory mechanisms that are implicated in the development of depression. Moreover, empirical data from preclinical investigations has demonstrated the influence of MIP-1β on mood-associated behaviors [32]. Based on these results, it appears that MIP-1β could potentially impact the regulation of mood and play a role in the development of depressive symptoms. The exact mechanisms through which MIP-1β affects mood and plays a role in the pathophysiology of MDD remain to be determined. Nevertheless, interactions with other inflammatory mediators, activation of microglia and astrocytes, disruption of neuroplasticity, and modulation of neurotransmitter systems, for instance, serotonin and glutamate, are all potential mechanisms by which MIP-1β influences mood [30, 32].

Macrophage chemoattractant protein-2 (MCP-2/CCL8) is a chemokine implicated in inflammation and immune responses [33]. Although research is scarce on MCP-2 for its specific association with MDD, emerging evidence indicates possible correlations between the dysregulation of MCP-2 and symptoms of depression [34, 35]. Dysregulation of the immune system and neuroinflammation have been proposed as contributing factors to the development of depressive symptoms. Notable studies have documented increased concentrations of MCP-2 in MDD, which could indicate that this chemokine may play a role in modulating the inflammatory mechanisms in depression [34]. Preclinical investigations have yielded knowledge regarding the possible function of MCP-2 in regulating mood. MCP-2 may influence mood-related behaviors and contribute to the pathophysiology of depression, according to these results [35]. The precise mechanisms through which MCP-2 may affect mood and contribute to MDD are uncertain.

The area of research that examining the role of chemokines such as MIP-1β and MCP-2 in MDD is important to understand the pathophysiology of depression. Although individuals with MDD exhibit elevated levels of these chemokines due to the onset of depressive symptoms, or elevated levels cause disorder [36, 37]. Therefore, this case-control investigation aimed to evaluate the role of MIP-1β and MCP-2 in the pathophysiology and development of MDD. Here, we planned to measure serum MIP-1β and MCP-2 levels in MDD patients compared to HCs and evaluate the strength of distinction ability of these chemokines as a diagnostic tool Also, we intended to compare the level of alterations with the severity of depressive symptoms to shed light on the underlying inflammatory processes that contribute to MDD and to pave the way for the development of diagnostic and therapeutic approaches that target neuroinflammatory pathways in MDD.

## 2. Methods and materials

### 2.1 Study population

This case-control study recruited 58 MDD patients and 30 HCs between September 1, 2023, and December 31, 2023. The MDD group consisted of individuals from a tertiary-level teaching hospital in Dhaka, Bangladesh, while the HCs were selected from different areas in Dhaka city. Age, sex, and sociodemographic characteristics were matched to ensure homogeneity between the two groups. The diagnosis of MDD and evaluation of symptom severity were based on the Diagnostic and Statistical Manual of Mental Disorders-5 (DSM-5) and the Hamilton rating scale for depression (Ham-D), respectively. Standard questionnaires were used to collect sociodemographic data and perform clinical assessments in the patient and HC groups. The authors had no access to information that could identify individual participants during or after data collection. The inclusion criteria were healthy individuals aged between 18 and 60 years. The excluded subjects from the present study who had Ham-D scores below eight, cognitive impairment, severe medical illness, history of kidney or liver failure, coexisting psychiatric disorders, and use of antipsychotic medications.

### 2.2 Sample collection

Each participant had 5 ml of blood drawn from their cephalic vein, which was left to clot in falcon tubes. The blood-containing tubes underwent centrifugation at a speed of 3000 rpm for around 15 minutes to obtain the serum. The serum was transferred to Eppendorf tubes and stored in a refrigerator at a temperature of -80°C to ensure optimal storage and preservation.

### 2.3 Measurement of serum MIP-1β and MCP-2

Commercially available enzyme-linked immunosorbent assay (ELISA) kits obtained from Boster Bio, USA, were employed to measure the serum levels of MIP-1β and MCP-2. To maintain consistency and to alleviate potential biases, all experiments were conducted by the same researchers, unaware of the outcomes, ensuring objective data analysis.

### 2.4 Statistical analysis

We used the Statistical Package for Social Sciences (SPSS) software version 25.0 along with Microsoft Excel to evaluate, sort, and statistical analysis of data. To differentiate between groups and explore the associations between variables, techniques such as independent samples t-tests, Chi-square testing, and correlation tests were employed. Spearman’s correlation analysis was used to examine the correlations among several study parameters in MDD patients. The introduction of boxplots facilitated the visualization of the provided data. Descriptive analysis was used to create socio-demographic profiles, and the findings were presented as the mean ± standard deviation (SD) with a 95% confidence interval (CI). The study effort used the receiver operating characteristic (ROC) curve to evaluate the diagnostic performance of biomarkers. Statistical significance was inferred for p-values equal to or less than 0.05.

### 2.5. Ethical consideration

The Research Ethics Committee of the University of Asia Pacific has approved the study protocol (UAP/REC/2023/208). Informed written consent was taken from all the attendees before data collection. The investigations were carried out in conformity with the principles outlined in the Helsinki Declaration.

## 3. Results

### 3.1 Description of the study population

The distribution of demographic profiles and a comparison between HCs and patients with MDD are displayed in Table 1. In both groups, the majority of individuals were between the ages of 18 and 25 (50.00% for MDD patients and 40.00% for HCs). Regarding the sex distribution, no statistically significant distinction was observed between MDD patients (male = 32.76% and female = 67.24%) and HCs (male = 30.00% and female = 70.00%) (p = 0.792). Regarding factors including marital status, educational level, occupation, economic status, area of residence, and smoking habit, there were no statistically significant disparities observed between MDD patients and the HCs (p>0.050). A family history of MDD was observed among 22.41% of MDD patients. In general, the HC group and the group of MDD patients exhibited comparable demographic characteristics.

**Table 1:** Demographic profile distribution and their comparison between MDD patients and healthy controls.

Parameters	MDD patients n (%)	Healthy controls n (%)	p-value
Age in years			0.857
18–25	29 (50.00)	12 (40.00)	
26–35	19 (32.76)	13 (33.34)	
36–45	8 (13.79)	4 (13.33)	
46–60	2 (3.45)	1 (3.33)	
Sex			0.792
Male	19 (32.76)	9 (30.00)	
Female	39 (67.24)	21 (70.00)	
BMI (kg/m^2^)			0.735
Below 18.5 (CED)	5 (8.62)	2 (6.67)	
18.5–25.0 (Normal)	42 (72.41)	21 (70.00)	
Above 25.0 (Obese)	11 (18.97)	7 (23.33)	
Marital status			0.635
Married	34 (58.62)	16 (53.33)	
Unmarried	24 (41.38)	14 (46.67)	
Education level			0.367
Primary	6 (10.34)	3 (10.00)	
Secondary	26 (44.83)	18 (60.00)	
Graduate and above	26 (44.83)	9 (30.00)	
Occupation			0.816
Business	8 (13.79)	4 (13.33)	
Service	8 (13.79)	2 (6.67)	
Housewife	21 (36.21)	10 (33.33)	
Unemployed	10 (17.24)	6 (20.00)	
Others	11 (18.97)	8 (26.67)	
Economic Status			0.274
Low	23 (39.66)	17 (56.67)	
Medium	22 (37.93)	7 (23.33)	
High	13 (22.41)	6 (20.00)	
Area of residence			0.914
Rural	20 (34.48)	10 (33.33)	
Urban	38 (65.52)	20 (66.67)	
Smoking History			0.318
Non-smoker	50 (86.21)	27 (90.00)	
Smoker	8 (13.79)	3 (10.00)	
Family history of MDD			0.002
Yes	13 (22.41)	2 (6.67)	
No	45 (77.59)	28 (93.33)	

Abbreviations: MDD, major depressive disorder, BMI, body mass index, CED, chronic energy deficiency.

### 3.2 Biophysical characteristics and laboratory findings

**Fig 1:**
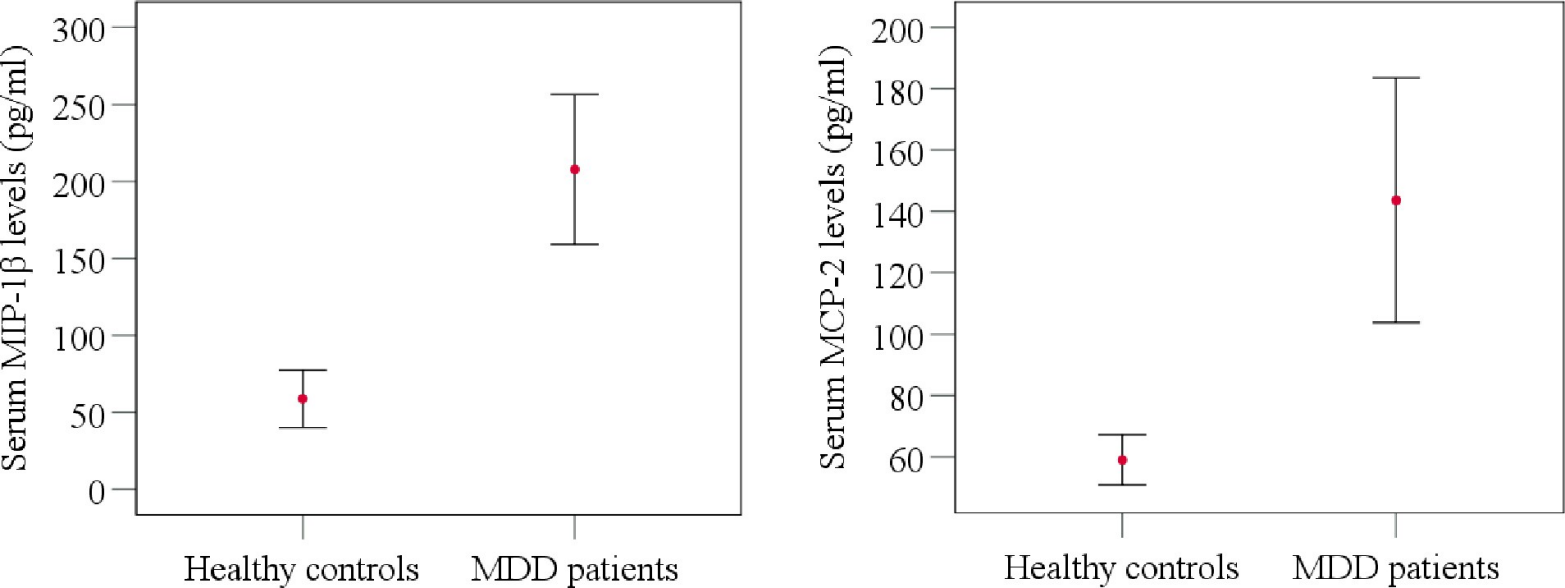
Comparison of serum MIP-1β and MCP-2 levels between MDD patients and healthy controls.

Table 2 presents the biophysical characteristics and laboratory findings of the study participants. The average age of MDD patients was 27.74±8.06years, whereas the age of HCs was 28.07±7.82 years; with no statistical significance (p = 0.857). MDD patients had a mean Body Mass Index (BMI) of 22.96±3.80 kg/m^2^, whereas HCs had a mean BMI of 23.25±3.91 kg/m^2^ (p = 0.735). MDD patients exhibited a substantially higher mean Ham-D score (18.57±5.73), in contrast to HCs (1.13±1.00; p<0.001). This finding suggests a substantial disparity in the manifestation of depressive symptoms between the two groups. Moreover, MDD patients exhibited significantly elevated serum levels of MIP-1β compared to HCs (207.73±184.63 pg/ml vs. 58.77±50.08 pg/ml, respectively) (p<0.001). In a similar vein, MDD patients exhibited significantly higher levels of MCP-2 in their serum in comparison to HCs (143.61±151.70 pg/ml vs. 56.84±21.99 pg/ml, respectively) (p = 0.003) (Fig 1).

**Table 2:** Clinical and laboratory findings of the study participants.

Parameters	MDD patients Mean ± SD (95% CI)	Healthy controls Mean ± SD ((95% CI)	p-value
Age in years	27.74±8.06 (2.12)	28.07±7.82 (2.92)	0.857
BMI (kg/m^2^)	22.96±3.80 (1.00)	23.25±3.91 (1.46)	0.735
Ham-D score	18.57±5.73 (1.51)	1.13±1.00 (0.37)	<0.001
MIP-1β (pg/ml)	207.73±184.63 (48.55)	58.77±50.08 (18.70)	<0.001
MCP-2 (pg/ml)	143.61±151.70 (39.89)	56.84±21.99 (8.21)	0.003

Abbreviations: BMI, body mass index; CI, confidence interval; MIP-1β, macrophage inflammatory protein-1β; MCP-2, monocyte chemoattractant protien-2; MDD, major depressive disorder; SD, standard deviation; Ham-D, Hamilton depression rating scale.

### 3.3 Correlation among the parameters

Table 3 presents Spearman’s correlation analysis results among various research parameters for MDD patients. A significant positive correlation was observed across the serum levels of MIP-1β and Ham-D scores (r = 0.451, p = 0.001) and serum concentrations of MCP-2 and Ham-D scores (r = 0.373, p = 0.004), respectively. This finding implies that elevated concentrations of these chemokines are linked to heightened severity of depressive symptoms. Additionally, a significant positive correlation (r = 0.292, p = 0.032) was identified between the concentrations of MCP-2 and MIP-1β, suggesting a possible association between these two chemokines in the pathogenesis of MDD.

**Table 3:** Spearman’s correlation study among various research parameters among MDD patients.

*Correlation parameters*	*r*	*p* *
Age and Ham-D score	-0.139	0.299
Age and MIP-1β	-0.115	0.390
Age and MCP-2	0.054	0.686
BMI and Ham-D score	-0.111	0.407
BMI and MIP-1β	0.030	0.820
BMI and MCP-2	-0.010	0.941
MIP-1β and Ham-D score	0.451	0.001
MCP-2 and Ham-D score	0.373	0.004
MIP-1β and MCP-2	0.292	0.032

Abbreviations: BMI, body mass index; MIP-1β, macrophage inflammatory protein-1β; MCP-2, monocyte chemoattractant protien-2; MDD, major depressive disorder; Ham-D, Hamilton depression rating scale.

*Bonferroni-corrected p values.

### 3.4 Receiver operating characteristic curve analysis

**Fig 2:**
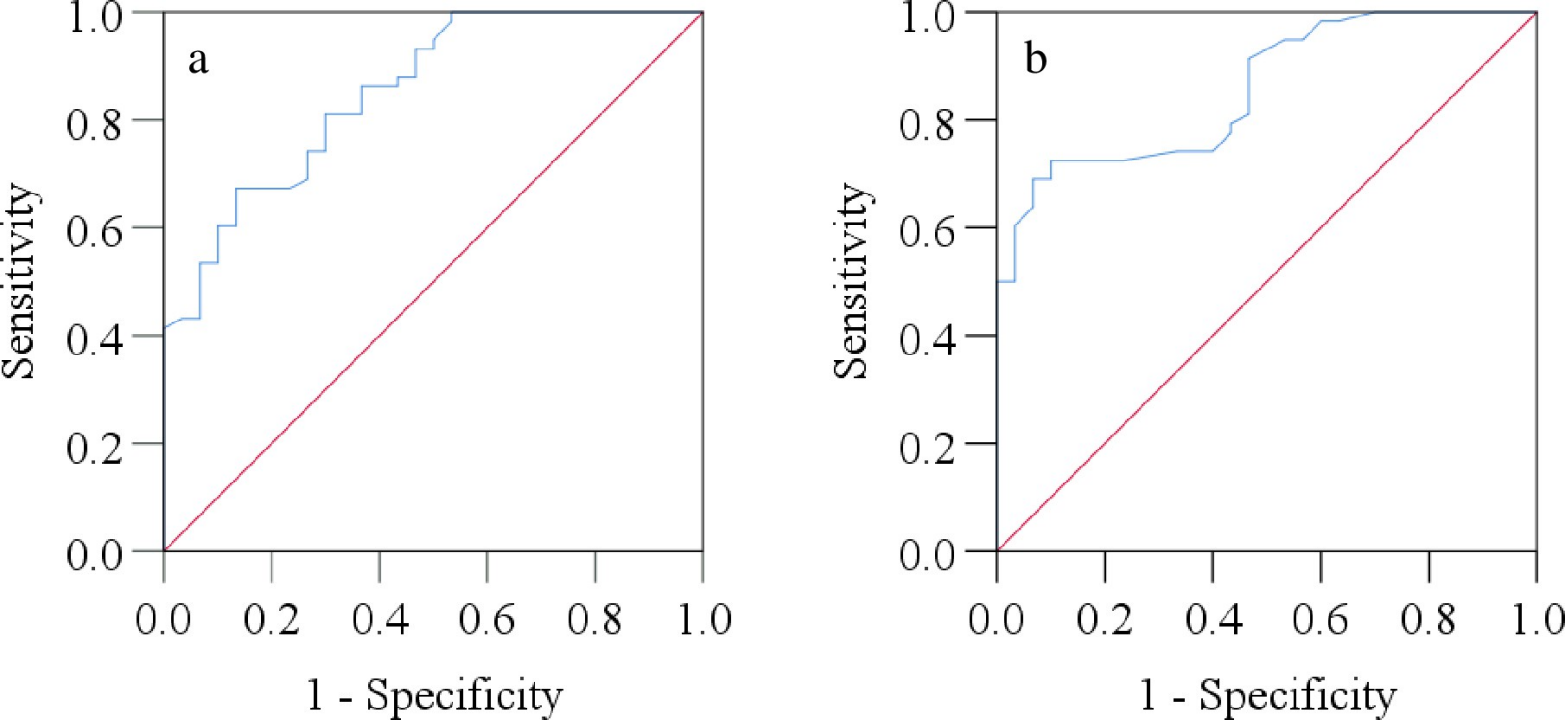
Receiver operating characteristic (ROC) curves of serum MIP-1β levels (a) and serum MCP-2 levels (b) among the study population.

The results of ROC curve analysis of serum MIP-1β and MCP-2 levels as potential diagnostic biomarkers for MDD are presented in Table 4 and Fig 2. The optimal cut-off value for discriminating between MDD patients and HCs was determined to be 63.18 pg/ml for MIP-1β and 60.85 pg/ml for MCP-2. At these cut-off values, MIP-1β exhibited a sensitivity of 82.8% and a specificity of 77.6%, with an Area Under the Curve (AUC) of 0.852 (95% CI: 0.771–0.932, p<0.001). Similarly, MCP-2 demonstrated a sensitivity of 84.4% and a specificity of 76.5%, with an AUC of 0.856 (95% CI: 0.780–0.933, p<0.001).

**Table 4:** Receiver operating characteristic (ROC) curve analysis of serum MIP-1β and MCP-2 levels.

Parameters	Cut-off value (pg/ml)	Sensitivity (%)	Specificity (%)	AUC	95% CI		p-value
					Lower bound	Upper bound	
MIP-1β	63.18	82.8	77.6	0.852	0.771	0.932	<0.001
MCP-2	60.85	84.4	76.5	0.856	0.780	0.933	<0.001

Abbreviations: AUC, area under the curve; CI, confidence interval; MIP-1β, macrophage inflammatory protein-1β; MCP-2, monocyte chemoattractant protien-2.

## 4. Discussion

This study found significantly higher levels of MIP-1β and MCP-2 in MDD patients than HCs, suggesting a potential association between these chemokines and MDD pathophysiology. Correlation analyses explored that higher chemokine levels were associated with increased severity of depressive symptoms. ROC curve analysis suggests that MIP-1β and MCP-2 has potential to be used as diagnostic biomarkers for MDD. Overall, these findings emphasize that elevated MIP-1β and MCP-2 levels may be associated with the pathophysiology and development of MDD. The present findings contribute to the existing understanding of the pathophysiology and mechanisms behind MDD [38–40]. Similar to earlier studies, these elevated levels of MIP-1β and MCP-2 in MDD indicate the involvement of neuroinflammatory processes in depression [29, 30, 34, 35]. Chemokines like MIP-1β and MCP-2 in the CNS are important for immune cell movement and inflammation. MIP-1β and MCP-2 are both proinflammatory chemokines, responsible for inducing and progression of inflammation. Although the understanding of the exact pathophysiology and pathways of initiation and development of MDD is still evolving, there is strong evidence that neuroinflammation contributes to the development of depressive symptoms [4, 25, 36, 41], as indicated by the significant positive correlation between levels of MIP-1β and MCP-2 and the severity of depressive symptoms. The study supports previous hypotheses that neuroinflammatory pathways play a crucial role in the development of MDD. It highlights the complex relationship between immune system dysregulation and brain function in depressive disorders [30, 34, 36, 42–45].

This study identified MIP-1β and MCP-2 as potential biomarkers associated with the severity of depressive symptoms. Additionally, the study shows that serum levels of MIP-1β and MCP-2 can differentiate between MDD patients and HCs, contributing to the advancement of objective diagnostic methods for MDD. Analysis of the ROC curve revealed that MIP-1β and MCP-2 can effectively differentiate between individuals with MDD and HCs with high sensitivity and specificity. This underscores the potential usefulness of these chemokines as precise diagnostic instruments, filling a crucial gap in psychiatric therapy since current diagnostic methods are predominantly based on subjective assessments. Establishing appropriate cut-off values and achieving high sensitivity and specificity of chemokines as diagnostic biomarkers are important to diagnose patients. To do this, physicians sometimes use objective metrics that can help in the early detection and diagnosis of MDD.

This study has significant implications for the diagnosis and treatment of MDD. Serum levels of MIP-1β and MCP-2 can be used as an early risk assessment tool for depressive symptoms. These may enable earlier intervention and therapy, improving patient outcomes. These biomarkers can help clinicians to screen for MDD risk factors or track symptom intensity and therapy response. When assessing and treating depressive patients, clinicians should also examine demographic parameters, other physical, behavioral, mental, clinical, and biochemical criteria for depression. This work provides quantitative diagnostic biomarkers and insights into the complexity of the condition of a patient in clinical practice. Clinicians can improve MDD diagnosis and management by using serum levels of MIP-1β and MCP-2 and considering many patient factors, leading to better patient outcomes and quality of life.

It is important to mention that the development and testing of biomarker panels consisting of MCP-2 and MIP-1β could improve the detection ability and accuracy of biomarker-based approaches used in the field of MDD. Researchers are exploring new and improved ways to predict and manage MDD. They are using a range of biomarkers, including neurochemicals, genetic markers, and neuroimaging investigations, to enhance predictive models and develop individualized and precision-driven strategies for managing MDD. Moreover, it is believed that targeting neuroinflammatory pathways through intervention studies aimed at regulating chemokine levels is crucial to testing the therapeutic potential of MDD. To advance the development of innovative approaches for MDD, researchers are also studying the effectiveness of immune-modulating therapies such as anti-inflammatory drugs, cytokine inhibitors, and other similar medications in reducing depressive symptoms and improving treatment results. Integrating data from psychiatry, neurology, genetics, and immunology can help achieve a comprehensive approach to the study of MDD. These can facilitate the development of personalized and targeted treatments for this debilitating condition. By considering these prospective methodologies, healthcare practitioners will sustain advancements in identifying, administering, and treating MDD.

Examination of demographic parameters, clinical manifestations, and serum concentration of biomarkers indicates the multifaceted nature of MDD. The application of established assessment instruments, such as the Ham-D, ensures standardization in the symptoms among participants, thereby strengthening the reliability of the results. Furthermore, the robust statistical methodologies supported the statistical reliability of the results. Nevertheless, the research is not devoid of limitations. The utilization of a cross-sectional design restricts the ability to establish causative associations between variables, thereby limiting the interpretation of results to associations solely. The concerns regarding the generalizability of the findings are due to the limited sample size which could be minimized in future studies by conducting on a larger population and by a more controlled population selection method. In addition, the potential confounding variables, including lifestyle choices and dietary patterns, were not thoroughly evaluated or accounted for, which could have affected the observed correlations. Therefore, further investigation is warranted, employing longitudinal designs, incorporating diverse samples, and conducting thorough assessments of confounding variables to effectively imply those findings in clinical practice.

The present study findings suggest several potential directions for the advancement of future research. These approaches provide possibilities to explore the underlying mechanisms of MDD and to advance diagnostic and therapeutic strategies. Firstly, we recommend longitudinal research to shed light on the fluctuations of chemokine expression and their relationship to the manifestation of depressive symptoms. To gain a better understanding of the relationship between neuroinflammation and the development of MDD, it is useful to investigate whether increased levels of MCP-2 and MIP-1β are present before the onset of depressive symptoms, or depression causes to elevate the levels. Further research is needed to investigate the mechanisms underlying the dysregulation of MCP-2 and MIP-1β in patients with MDD. A better comprehension of the complex link between immunological dysregulation and neuronal function in depressive disorders can be achieved through researching the interactions among these chemokines, neurobiological pathways, environmental factors, and other inflammatory mediators. Additionally, investigating the influence of genetic and epigenetic factors on chemokine expression may offer better management for MDD.

## 5. Conclusion

The present study results suggest the potential association of MIP-1β and MCP-2 with the pathophysiology of MDD development. The high levels of these chemokines in the blood could be used as markers for the severity of depression symptoms, helping in more precise diagnosis and tailored treatment strategies. In the future, longitudinal studies are necessary to clarify the actual role of these chemokine expression on the progression of MDD. Moreover, investigating the fundamental causes of chemokine dysregulation and creating multiplex biomarker arrays could improve diagnostic precision and treatment strategies. However, the current study has limitations in sample size and lack of grip on different confounding variables, which could be minimized in future studies by conducting on a larger population and by a more controlled population selection method. Considering cytokine and chemokine dysregulation could transform the diagnosis and treatment of MDD, resulting in more efficient treatment approaches and better outcomes for individuals with this severe disorder.

## Supporting information

S1 Checklist (DOCX)
